# Bacterial toxicity of Acetaminophen and Edaravone, and their binary mixtures: experimental and predicted values using traditional and novel Van Laar-based models

**DOI:** 10.1007/s10646-024-02772-w

**Published:** 2024-06-29

**Authors:** Iván Álvarez-Escalante, Sonia Martínez-Páramo, Rubén Irusta-Mata

**Affiliations:** 1 Department of Chemical Engineering and Environmental Technology, University of Valladolid, Calle Doctor Mergelina s/n, 47011 Valladolid, Spain; 2 Institute of Sustainable Processes, University of Valladolid, Calle Doctor Mergelina s/n, 47011 Valladolid, Spain

**Keywords:** Microtox®, Pharmaceuticals, Binary mixtures, Concentration Addition, Independent Action, Van Laar-based model

## Abstract

In recent years, the presence of Pharmaceutical Active Compounds (PhACs) in ecosystems has become a serious environmental problem due to their capacity to induce harmful effects at extremely low concentrations in both humans and wildlife. Water treatment plants have not been designed to remove these types of compounds efficiently. Thus, the detection of these pollutants is essential to evaluate their negative impacts and is one of the emerging issues in environmental chemistry. The main objective of this study is to determine the bacterial toxicity of two PhACs (both individually and as a mixture) through the quantification of bioluminescence inhibition in the marine bacteria *Aliivibrio fischeri*, a commonly used method in short-term toxicity tests. In this work, Acetaminophen and Edaravone, two drugs approved by the Food and Drug Administration, have been studied. The acute toxicity of these PhACs has been tested at two exposure times (5 and 15 min) and different concentrations, by estimation of the median effective concentration (EC_50_) for each individual compound or in combination at different concentrations. Moreover, the EC_50_ of the binary mixtures Acetaminophen/Edaravone have been forecast using two traditional predictive models, Concentration Addition and Independent Action. The results show that toxicity decreases with exposure time and depends on the concentration tested. Furthermore, a novel semi-empirical Van Laar-based model has been proposed and validated with the experimental data from this study and literature data, obtaining satisfactory estimations of the EC_50_ for binary mixtures.

## Highlights

- The bacterial toxicity of Acetaminophen and Edaravone, as well as their binary mixtures, was evaluated using an *Aliivibrio fischeri* bioluminescence assay.
- The predictive power of Concentration Addition and Independent Action models was evaluated for combined bacterial toxicity of PhACs.
- A new semi-empirical Van Laar-based model was developed and validated to predict the bacterial toxicity of binary mixtures of Acetaminophen and Edaravone, providing accurate estimations with limited experimentation.

## Introduction

Nowadays, the presence of Active Pharmaceutical Compounds (PhACs) in ecosystems has become a serious environmental problem due to their capacity to induce harmful effects in both humans and wildlife even at extremely low concentrations (dos Santos et al. 2021). The high consumption of these substances in households and hospitals exacerbates their environmental impact (Peake et al. 2016; Ebele et al. 2017).

Toxicity refers to the ability of a chemical substance to induce harmful effects in an organism. Ecotoxicity, on the other hand, measures the toxic effect resulting from the presence of a chemical compound in an ecosystem. It is described in terms of the exposure time and concentration of the hazardous substance. Thus, acute ecotoxicity represents the harmful effects on ecosystem constituents during a short exposure time, typically less than the reproductive cycle of the most sensitive test organism, while chronic ecotoxicity quantifies the effects over a longer duration, usually spanning from the reproductive cycle to a certain number of years, depending on the test species. (Waller and Allen 2008).

Standard toxicity tests are used to assess the effect of chemical compounds on organisms such as algae, crustaceans, fish, and microorganisms like bacteria. An example of this is the bioluminescence inhibition assay of the bacteria *Aliivibrio fischeri*, which has been widely applied in the literature (Ortiz de García et al. 2014). Hence, toxicity bioassays were performed using the aforementioned microorganism with a Microtox® assay in the present work. This assay has been extensively validated due to its high sensitivity, particularly compared to tests that assess organisms of high complexity (Abbas et al. 2018).

Microtox® possesses numerous applications as a result of its capacity to analyze a wide range of analytes, including atmospheric pollutants, pesticides, and nanoparticles. It is mostly employed in two domains: industrial effluents and wastewater evaluation. In the former, it has been validated as an efficient test for analyzing effluents from multiple industries, such as pharmaceutical, food, and textile. In the latter, it is used to study the removal of contaminants from wastewater (Abbas et al. 2018). However, numerical values are essential to determine the pollution potential of the contaminant. Therefore, toxicological parameters – such as the median effective concentration (EC_50_) – i.e. the concentration of a substance expected to produce a certain effect in 50% of the tested organisms – have been developed for this purpose.

Researchers from numerous countries have detected dozens of PhACs and their metabolites in different aquatic environments (wastewater, ground water, etc.). Many of these compounds are being introduced into ecosystems undetected (Gogoi et al. 2018). Moreover, most PhACs are not fully eliminated through wastewater treatment and do not naturally degrade in the environment (Heberer 2002; Wilkinson et al. 2017; Liu et al. 2018; Couto et al. 2019). Although many studies in the literature have examined the ecotoxicity of a variety of PhACs used in different therapeutic classes (Maranho et al. 2015; Ortiz de García et al. 2016; He et al. 2018), it is necessary to carry out ecotoxicological studies on newer compounds.

In 2017, the FDA approved Edaravone (EDA) (Radicava, Mitsubishi Tanabe Pharma America) (Table S1), a PhAC consisting of a free radical scavenger that aims to slow down dysfunction in patients with amyotrophic lateral sclerosis (ALS) (Cruz 2018). No well documented ecotoxicological studies have been conducted on this drug to date. Therefore, it is crucial to evaluate EDA’s ecotoxicity.

However, aquatic organisms are typically exposed not to just one type of substance but to numerous compounds at the same time. As a result, it is necessary to experimentally investigate the combined ecotoxicity of a binary mixture containing EDA and the widely-used analgesic and antipyretic Acetaminophen (ACM) (Table S1). Given that ACM is one of the most commonly consumed drugs in Spain (Ortiz de García et al. 2013), it is quite likely that it will be administered jointly with EDA.

Nevertheless, in most cases, it is not feasible to experimentally measure the ecotoxicity of multicomponent mixtures since the corresponding tests are arduous and time-consuming. Therefore, a common alternative is to estimate the overall ecotoxicity of the mixture from the individual ecotoxicities, using mathematical models (Backhaus et al. 2000). Numerous models for estimating mixture ecotoxicity have been introduced in the literature, with the most popular being Concentration Addition (CA) and Independent Action (IA), also known as Loewe additivity (Loewe 1926) and Bliss independence (Bliss 1939), respectively.

CA model assumes that all the components of the mixture have the same mechanism of action, interfere physiologically at the same molecular target site, and do not interact with each other (Wang et al. 2020). This model is generally accepted as a solution for ecotoxicity analysis in mixtures (Altenburger et al. 2000).

The second model, IA, assumes that the mechanism of action of every compound is different and that each drug acts in a site of action that has not been affected by other components (Wang et al. 2020). Like the CA model, this model shares the assumption that the components do not interact with each other (Faust et al. 2003).

On the other hand, it is well known in Chemical Engineering that certain properties of a binary mixture in vapor-liquid equilibria can be estimated using an activity coefficient correlation, provided that there is experimental data to compute the binary interaction parameters that are present in this correlation (Henley and Seader 1981). A well-known example that uses activity coefficients is the Van Laar equation (Van Laar 1906). Thus, by establishing an analogy with a vapor-liquid equilibrium, a new Van Laar-based semi-empirical model has been proposed and validated using the experimental data from this work and literature data. This model arises from the need to save both time and effort in experimental tests, which even in mixtures with few components are extremely arduous. In fact, this novel model requires only one experimental point to carry out an estimation, unlike other ecotoxicology models in the literature that require several experimental points to make predictions.

Therefore, the main objective of this work was to experimentally determine the bacterial toxicity of ACM and EDA and their binary mixtures, as well as to predict the toxicity of these binary mixtures using theoretical and semi-empirical models.

Results were assessed using the Combination Index (CI) method, which quantifies deviations from additivity and is widely utilized in the literature (Chou 2006).

## Materials and methods

### Selection of the PhACs

It is worth noting that EDA and ACM share some similarities in their molecular structures, particularly in their functional groups. Specifically, both compounds contain a carbonyl group and a benzene ring. Furthermore, their molecular weights are numerically comparable, as shown in Table S1. Additionally, there is a high probability that both PhACs coexist in the aquatic environment, as ACM ranks among the most commonly consumed drugs in Spain (Ortiz de García et al. 2013).

Current research on the environmental presence of EDA is limited. Conversely, some studies have documented the presence of ACM in wastewater effluents. Wu et al. (2023) reported concentrations as high as 0.09 mg L^−1^, while Petrie et al. (2015) and Hughes et al. (2013) identified concentrations of 0.01 and 0.02 mg L^−1^, respectively. These findings suggest that ACM may pose a potential ecological risk, warranting further investigation.

#### Edaravone

Edaravone, with the chemical name of 3-methyl-1-phenyl-2-pyrazolin-5-one, appears as a white crystalline compound and has a melting point of 129.7 °C (Table S1). It is soluble in some organic compounds and slightly soluble in water. Following intravenous administration, 1% of the dose is excreted without any alteration, and between 71 and 79.9% is eliminated in the form of metabolites, predominantly glucuronide and sulfate conjugates (Cruz 2018). Notably, no ecotoxicological studies have been conducted on this drug in the USA and EU to date. The Academic Canadian repository DrugBank (DrugBank Online 2024) does provide Safety Data Sheets containing ecotoxicity data, but the source’s verifiability is compromised due to the absence of OECD test guidelines or GLP listing.

#### Acetaminophen

Acetaminophen, also known as Paracetamol, is an analgesic and antipyretic drug with the chemical name N-(4-hydroxyphenyl) acetamide. It appears as a white crystalline solid at room temperature and has a melting point of 169 °C. The drug is soluble in water (Table S1). Its individual ecotoxicity has been extensively studied in previous research (Calleja et al. 1993; Henschel et al. 1997; Kim et al. 2007; Ortiz de García et al. 2014; Phong Vo et al. 2019).

### Chemicals

Analytical or technical grade PhACs with a purity of > 95% were obtained from Sigma-Aldrich in order to carry out the toxicity tests:

- 1-Phenyl-3-methyl-2-pirazolin-5-one (Edaravone): purity 99% Sigma-Aldrich (Ref. M70800 ALDRICH).
- Acetaminophen (Paracetamol): BioXtra, purity 99% Sigma-Aldrich (Ref. A7085 SIGMA-ALDRICH).

### Test organisms and media

To evaluate acute toxicity, Microtox® tests were performed using the marine bioluminescent bacterium *A. fischeri* as the test organism. The bacterium was obtained from Modern Water Inc. USA in a freeze-dried form and stored at −25 to −20 °C to maintain microbial activity. All solutions of pharmaceuticals and personal care products were prepared using Milli-Q® water. Additionally, two solutions of NaCl (2 and 22% w/v) were utilized as a saline medium and for achieving osmotic equilibrium, respectively. Furthermore, both compounds have been carefully maintained in their nonionized forms, as the pH range during the experiments is tightly controlled between 6 and 8. As a result, any potential effects of ionized compounds on toxicity can be disregarded.

### Bacterial toxicity tests

#### Fundamentals

A bioluminescence assay is a technique that involves studying the variation of light emitted by a bioluminescent marine bacterium, *A. fischeri* (formerly known as *Vibrio fischeri* or *Photobacterium phosphoreum*). This bacterium is highly sensitive to a wide range of toxic compounds. When exposed to a toxic substance, the bacteria respond by reducing their luminescence. In other words, the intensity of light emitted decreases as the toxicity of a substance increases.

Microtox® is a widely used bioluminescence assay developed by Strategic Diagnostic Inc. (AZUR Environmental). In this study, the test was performed according to the manufacturer’s recommended procedure (ISO 11348-3 standard) and the user manual (Microbics Corporation 1995). This method offers several advantages, including its reliability, speed, low cost, and high sensitivity. Additionally, the equipment required for the test is compact and minimal sample is needed. Moreover, the ethical concerns related to experiments involving more complex organisms, such as fish and rats, are avoided when using microorganisms. Finally, it is worth noting that all experiments were conducted at least twice to ensure reproducibility and obtain acceptable confidence intervals.

#### Calculations and errors associated to the bioluminescence test

As previously mentioned, the degree of toxicity is evaluated based on the EC_50_ of the tested substance, which causes a 50% reduction in the luminosity of the bacteria. To assess the changes in bioluminescence of the *A. fischeri* bacteria exposed to toxic compounds, the relative variation in light intensity emitted by the bacteria (Γ) was calculated for each concentration at a specific exposure time (t) of the tested substance. The value of Γ is defined as the ratio of the loss of light intensity to the emitted light (I_t_) measured in luminescence units at a given instant (Eq. 1).

1 $$\Gamma =\frac{{\rm{BR}}\bullet {{\rm{I}}}_{0}-{{\rm{I}}}_{{\rm{t}}}}{{{\rm{I}}}_{{\rm{t}}}}$$

where BR is a correction factor that represents the ratio between the intensity in the absence of a toxic substance and the initial intensity I_0_. This correction factor is introduced to account for the natural light reduction throughout the test.

Moreover, the toxic effect (E(%)) of the chemical on the bioluminescence of the bacteria can be defined as a function of the parameter Γ (Eq. 2).

2 $${\rm{E}}( \% )=\frac{\Gamma }{1+\Gamma }\bullet 100$$

After obtaining the function Γ vs C (where C represents the concentration of the toxic compound/s being tested), a logarithmic transformation can be applied to linearize it. This allows expressing it in terms of log(Γ) vs log(C), and obtaining the fitting line of Eq. 3.

3 $$\log \left(\Gamma \right)={\rm{A}}\bullet \log \left({\rm{C}}\right)+{\rm{B}}$$

where A and B correspond to linear regression parameters. The EC_50_ can be determined by applying Γ = 1 to Eq. 3, which leads to Eq. 4.

4 $${{\rm{EC}}}_{50}={10}^{-{\rm{B}}/{\rm{A}}}$$

The toxicity results obtained for the EC_50_ can be classified according to the Globally Harmonized System of Classification and Labeling of Chemicals (GHS) established by the United Nations in 2011. The categories defined by this system are as follows:

- Highly toxic: EC_50_ ≤ 1 mg L^−1^
- Toxic: 1 mg L^−1^ < EC_50_ ≤ 10 mg L^−1^
- Harmful to aquatic organisms: 10 mg L^−1^ < EC_50_ ≤ 100 mg L^−1^
- Non-toxic: EC_50_ > 100 mg L^−1^

Regarding the calculation of EC_50_ values using the Microtox® method, it is important to consider potential errors. One way to account for uncertainty is to compute the confidence interval of the log (EC_50_) value, which can be achieved using Eq. 5. This equation is based on the method described by Irusta et al. (2011) for a confidence level of 95%.

5 $${\log \Gamma }_{{\rm{EC}}50}\pm {{\rm{t}}}_{(0.025,{\rm{N}}-2)}\bullet {{\rm{S}}}_{{\rm{yx}}}\bullet \sqrt{\left[\frac{1}{{\rm{N}}}+\frac{{\left({\log {\rm{EC}}}_{50}-\overline{\log {\rm{C}}}\right)}^{2}}{\mathop{\sum }\nolimits_{1}^{{\rm{N}}}{\left({\log {\rm{C}}}_{{\rm{i}}}-\overline{\log {\rm{C}}}\right)}^{2}}\right]}$$

where the residual standard deviation (S_yx_) is calculated using Eq. 6, where N is the total number of tests, EC_50_ is expressed in mg L^−1^, Γ_EC50_ is its corresponding Γ value, and t_(0.025, N-2)_ is the Student’s t distribution value at a confidence level of 95% with N-2 degrees of freedom.

6 $${{\rm{S}}}_{{\rm{yx}}}^{2}=\left[\frac{1}{{\rm{N}}-1}\right]\left[\mathop{\sum }\limits_{1}^{{\rm{N}}}(\overline{\log \Gamma }-{\log \Gamma }_{{\rm{i}}})^{2}-\frac{{\left[\mathop{\sum }\nolimits_{1}^{{\rm{N}}}(\overline{\log \Gamma }-{\log \Gamma }_{{\rm{i}}})\bullet (\overline{\log {\rm{C}}}-{\log {\rm{C}}}_{{\rm{i}}})\right]}^{2}}{\mathop{\sum }\nolimits_{1}^{{\rm{N}}}{\left(\overline{\log {\rm{C}}}-{\log {\rm{C}}}_{{\rm{i}}}\right)}^{2}}\right]$$

where Γ_i_ is the value of Γ for a test i, $\overline{{\mathrm{log}}\; \Gamma}$ is the average value of log Γ_i_, $\overline{\log {\rm{C}}}$ is the average value of log C_i_, and C_i_ represents the PhAC or the PhAC mixture for a certain test i.

Regarding the errors associated with the calculation of the toxic effect (Eq. 2), we used the error propagation method as described by Taylor (1982). To compute the errors, we calculated the prediction interval following Eqs. 7, 8, and 9 (Irusta et al. 2011).

7 $${\rm{\delta}} \; \log{\Gamma }_{{\rm{j}}}={{\rm{t}}}_{(0.025,{\rm{N}}-2)}\bullet {{\rm{S}}}_{{\rm{yx}}}\bullet \sqrt{\left[\frac{1}{{\rm{N}}}+\frac{{\left({\log {\rm{C}}}_{{\rm{j}}}-\overline{\log {\rm{C}}}\right)}^{2}}{\mathop{\sum }\nolimits_{1}^{{\rm{N}}}{\left({\log {\rm{C}}}_{{\rm{i}}}-\overline{\log {\rm{C}}}\right)}^{2}}\right]}$$

8 $${\rm{\delta}} {\Gamma }_{\rm{j}}=\frac{{\rm{\delta}} \;{\rm{log}}\,{\Gamma }_{{\rm{j}}}}{{\left|\frac{{\rm{d}}\,{\log}\,\Gamma }{{\rm{d}}\Gamma }\right|}_{\rm{j}}}=\left|{\Gamma }_{\rm{j}}\right|{{\cdot }}{\mathrm{ln}}10{{\cdot }}{\rm{\delta}} \; {\log}\,{\Gamma }_{{\rm{j}}}$$

9 $${\rm{\delta}} {{\rm{E}}( \% )}_{\rm{j}}={\left|\frac{{{\rm{d}}{\rm{E}}}( \% )}{{\rm{d}}\Gamma }\right|}_{\rm{j}}{{\cdot }}{\rm{\delta}} {\Gamma}_{\rm{j}}=\frac{1}{{\left(1+{\Gamma }_{\rm{j}}\right)}^{2}}{{\cdot }}{\rm{\delta}} {\Gamma}_{\rm{j}}$$

where δ log Γ_j_ corresponds to the error of the confidence interval of $\log {\Gamma }_{{\rm{j}}}$, C_j_ is the concentration of PhAC or the PhAC mixture in mg L^−1^, t_(0.025, N-2)_ is the value of the Student’s t distribution for a confidence level of 95% with N-2 degrees of freedom, and Γ_j_ is the value of Γ for the PhAC´s concentration C_j_.

### Estimation of the toxicity in mixtures with mathematical models

Experimental studies have demonstrated that for mixtures of substances with known mechanisms of action, both the traditional CA and IA models are valid only if their respective hypotheses are met (Altenburger et al. 2005). However, from a biological perspective, the toxic effects on bacteria are much more complex (Carbajo et al. 2015). As a result, secondary modes of action may exist, which are not considered by these two models. Moreover, the existence of interactions that affect global toxicity has been demonstrated (Rider and LeBlanc 2005). Despite the usefulness of the CA and IA models in predicting toxicity of mixtures, their applicability can be limited due to the assumptions underlying these models. Specifically, in the natural environment, mixtures may contain various compounds that do not conform to the IA and CA models hypotheses (Olmstead and LeBlanc 2005), which may restrict their predictive power. Nevertheless, some researchers argue that the assumptions of these models are not always closely linked to their prediction accuracy (Godoy et al. 2019; Yang et al. 2017; Wang et al. 2020). Therefore, despite the diversity in the mechanisms of action, IA and CA can still serve as viable models for estimating mixture toxicity.

Several authors have developed models to evaluate toxicity in a more precise manner by integrating the CA and IA models. The Integrated Addition and Interaction (IAI) model presented by Rider and LeBlanc (2005) and Olmstead and LeBlanc (2005) combines the concepts of CA and IA by assigning substances with the same mechanism of action to a group and calculating their global toxicity using the CA model, while estimating the toxicity of compounds with different mechanisms using the IA model. The Two Stage Prediction (TSP) model uses a similar integrative approach, but the prediction is divided into two stages (Mo et al. 2017). In contrast, Qin et al. (2011) proposed the Integrated Concentration Addition with Independent Action (ICIM) model, which, like the IAI and TSP models, utilizes the CA and IA models but integrates them with multiple linear regression.

#### Concentration addition model

The EC_50_ of a binary mixture, (EC_50_)_mix,CA_, can be determined using the CA model (Loewe 1926) by Eq. 10, which takes into account the experimental values of the individual EC_50_ of each component j, (EC_50_)_j,exp_.

10 $${\left({{\rm{EC}}}_{50}\right)}_{{\rm{mix}},{\rm{CA}}}={\left(\mathop{\sum }\limits_{{\rm{j}}=1}^{2}\frac{{{\rm{w}}}_{{\rm{j}}}}{{{({\rm{EC}}}_{50})}_{{\rm{j}},\mathrm{exp}}}\right)}^{-1}$$

where ${{\rm{w}}}_{{\rm{j}}}$ gives the mass fraction of compound j, (EC_50_)_mix,CA_ is the EC_50_ estimated by the CA model in mg L^−1^, and (EC_50_)_j,exp_ corresponds to the experimental individual EC_50_ of compound j in mg L^−1^.

#### Independent action model

In accordance with the IA model proposed by Bliss (1939), the toxicological effect of a binary mixture with a concentration of c_mix_ can be described by Eq. 11.

11 $${\rm{E}}\left({{\rm{c}}}_{{\rm{mix}}}\right)=1-\mathop{\prod }\limits_{{\rm{j}}=1}^{2}\left(1-{{\rm{E}}}_{{\rm{j}}}\left({{\rm{c}}}_{{\rm{j}}}\right)\right)$$

Here, ${{\rm{E}}}_{{\rm{j}}}\left({{\rm{c}}}_{{\rm{j}}}\right)$ represents the effect of an individual compound j at concentration c_j_, and ${\rm{E}}\left({{\rm{c}}}_{{\rm{mix}}}\right)$ is the global effect of the mixture at concentration c_mix_. ${{\rm{E}}}_{{\rm{j}}}\left({{\rm{c}}}_{{\rm{j}}}\right)$ can be expressed as a function of the mass fractions of the compounds in the mixture ${{\rm{E}}}_{{\rm{j}}}\left({{\rm{w}}}_{{\rm{j}}}\bullet {{\rm{c}}}_{{\rm{mix}}}\right)$. Assuming that ${\rm{E}}\left({{\rm{c}}}_{{\rm{mix}}}\right)=0.5$, the total concentration of the compounds can be calculated as c_mix_ = (EC_50_)_mix, IA_. Hence, Eq. 12 is derived as follows:

12 $$1-\,\mathop{\prod }\limits_{{\rm{j}}=1}^{2}\left(1-{\rm{E}}_{\rm{j}}\left({\rm{w}}_{\rm{j}}\bullet {\left({\rm{EC}}_{50}\right)}_{{\rm{mix}},\,{\rm{IA}}}\right)\right)=0.5$$

Equation 12 implies that (EC_50_)_mix,IA_ cannot be directly determined and requires iterative methods for its approximation, as noted by Faust et al. (2003). Additionally, the equation highlights the need for a mathematical expression of the Concentration-Effect curve for each component, ${{\rm{E}}}_{{\rm{j}}}\left({{\rm{c}}}_{{\rm{j}}}\right)$, to apply the IA model. In the literature, this curve is often modeled by a Weibull distribution, F_j_(c_j_) (Eq. 13) (Villa et al. 2014).

13 $${{\rm{F}}}_{{\rm{j}}}\left({{\rm{c}}}_{{\rm{j}}}\right)={{\rm{E}}}_{{\rm{j}}}\left({{\rm{c}}}_{{\rm{j}}}\right)=1-\mathrm{exp}\left[-\exp \left({\rm{\alpha }}+{\rm{\beta }}\bullet \log \left({{\rm{c}}}_{{\rm{j}}}\right)\right)\right]$$

where α and β are model parameters, c_j_ is the concentration of an individual compound, and E_j_ represents the inhibitory effect caused by the compound.

#### The new proposed Van Laar-based model

There are various toxicology models available in the literature that aim to estimate the toxicity of mixtures at different doses. These models can generally be classified as either predictive or empirical. Predictive models, such as the traditional CA and IA models and their combinations, do not require any experimental data on the toxicity of the mixture. On the other hand, empirical models simply perform a regression of the experimental data, such as the ICIM model. In this study, a semi-empirical model based on Van Laar equations has been proposed to save time and resources as it would only require one measurement for estimation.

When applying thermodynamics to vapor-liquid equilibrium, the goal is to estimate the temperature, pressure, and composition of each phase. This can be accomplished using models that describe the behavior of vapor-liquid equilibrium (VLE) systems (Smith et al. 2001). One such model is Raoult’s law (Raoult 1887), which is used to predict VLE in ideal solutions. It is the simplest and most well-known model, and is represented by Eq. 14.

14 $${{\rm{y}}}_{{\rm{i}}}=\left(\frac{{{\rm{P}}}_{{\rm{i}}}^{{\rm{sat}}}}{{{\rm{P}}}_{{\rm{T}}}}\right){{\rm{x}}}_{{\rm{i}}}$$

where x_i_ is the mole fraction of the liquid phase, y_i_ is the mole fraction of the vapor phase, ${{\rm{P}}}_{{\rm{i}}}^{{\rm{sat}}}$ is the vapor pressure of pure component i, and P_T_ is the total pressure of the system.

Similarly, it might be possible to apply this principle to toxicology and predict the toxicity of an ideal binary mixture, ${\left({{\rm{EC}}}_{50}\right)}_{{\rm{mix}}}^{* }$, using only the mass fraction w_j_ of each component and its individual experimental toxicity (EC_50_)_j,exp_ (Eq. 15).

15 $${\left({{\rm{EC}}}_{50}\right)}_{{\rm{mix}}}^{* }\,=\,\mathop{\sum }\limits_{{\rm{j}}=1}^{2}{{({\rm{EC}}}_{50})}_{{\rm{j}},\mathrm{exp}}\bullet {{\rm{w}}}_{{\rm{j}}}$$

However, it is well-known that the toxic effect of different PhAC mixtures does not follow linear or ideal behavior (Boillot and Perrodin 2008; Escher et al. 2010), and additive, synergistic and antagonistic responses are present (Villa et al. 2014; González-Pleiter et al. 2013; Yang et al. 2017; Dong et al. 2019; Ukić et al. 2019). Therefore, a linear estimation such as Eq. 15 needs to be corrected to account for this behavior. In thermodynamics, this correction can be achieved using the Van Laar equation, which includes activity coefficients ${{\rm{\gamma }}}_{{\rm{j}}}$ as parameters. Equation 16 shows the Van Laar equation under low pressure assumption and with the vapor-phase being an ideal gas mixture (Smith et al. 2001).

16 $${{\rm{y}}}_{{\rm{j}}}={{\rm{\gamma }}}_{{\rm{j}}}\left(\frac{{{\rm{P}}}_{{\rm{j}}}^{{\rm{sat}}}}{{{\rm{P}}}_{{\rm{T}}}}\right){{\rm{x}}}_{{\rm{j}}}$$

where the activity coefficients ${{\rm{\gamma }}}_{{\rm{j}}}$ for a binary mixture can be calculated using the binary interaction parameters A_12_ and A_21_, which are presented in Eqs. 17 and 18, respectively.

17 $$\mathrm{ln}\left({{\rm{\gamma }}}_{1}\right)={{\rm{A}}}_{12}{\left[\frac{{{\rm{A}}}_{21}{{\rm{w}}}_{2}}{{{\rm{A}}}_{12}{{\rm{w}}}_{1}+{{\rm{A}}}_{21}{{\rm{w}}}_{2}}\right]}^{2}$$

18 $$\mathrm{ln}\left({{\rm{\gamma }}}_{2}\right)={{\rm{A}}}_{21}{\left[\frac{{{\rm{A}}}_{12}{{\rm{w}}}_{1}}{{{\rm{A}}}_{12}{{\rm{w}}}_{1}+{{\rm{A}}}_{21}{{\rm{w}}}_{2}}\right]}^{2}$$

Thus, similar to the correction of Raoult’s law with the Van Laar equation in the VLE, this study proposes toxicological activity coefficients, ${{\rm{\gamma }}}_{{\rm{j}}}^{{\prime} }$, which transform Eq. 15 into Eq. 19. The purpose of this transformation is to estimate the EC_50_ using a Van Laar-based model.

19 $${\left({{\rm{EC}}}_{50}\right)}_{{\rm{mix}},{\rm{VL}}}=\,\mathop{\sum }\limits_{{\rm{j}}=1}^{2}{{{{\rm{\gamma }}}_{{\rm{j}}}^{{\prime} }\bullet ({\rm{EC}}}_{50})}_{{\rm{j}},\mathrm{exp}}\bullet {{\rm{w}}}_{{\rm{j}}}$$

where the EC of the mixture calculated with the Van Laar-based model is denoted by ${\left({{\rm{EC}}}_{50}\right)}_{{\rm{mix}},{\rm{VL}}}$, where ${{({\rm{EC}}}_{50})}_{{\rm{j}},\mathrm{exp}}$ represents the experimental EC_50_ of individual compound j. The toxicological activity coefficient is denoted by ${{\rm{\gamma }}}_{{\rm{j}}}^{{\prime} }$ and w_j_ is the mass fraction of compound j. For a binary mixture, the ${{\rm{\gamma }}}_{{\rm{j}}}^{{\prime} }$ values can be computed using Eqs. 20 and 21, which are analogous to Eqs. 17 and 18 and are given below.

20 $${\mathrm{ln}}\left({{\rm{\gamma }}}_{1}^{{\prime} }\right)={\rm{A}}_{12}^{ \acute{} }{\left[\frac{{\rm{A}}_{21}^{ \acute{} }{{\rm{w}}}_{2}}{{\rm{A}}_{12}^{ \acute{} }{{\rm{w}}}_{1}+{\rm{A}}_{21}^{ \acute{} }{{\rm{w}}}_{2}}\right]}^{2}$$

21 $${\mathrm{ln}}\left({{\rm{\gamma }}}_{2}^{{\prime} }\right)={\rm{A}}_{21}^{ \acute{} }{\left[\frac{{\rm{A}}_{12}^{ \acute{} }{{\rm{w}}}_{1}}{{\rm{A}}_{12}^{ \acute{} }{{\rm{w}}}_{1}+{\rm{A}}_{21}^{ \acute{} }{{\rm{w}}}_{2}}\right]}^{2}$$

Equations 20 and 21 show how the toxicological activity coefficients ${{\rm{\gamma }}}_{{\rm{j}}}^{{\prime} }$ for a binary mixture are computed using the Van Laar-based model. Specifically, ln(${{\rm{\gamma }}}_{1}^{{\prime} }$) and ln(${{\rm{\gamma }}}_{2}^{{\prime} }$) are calculated using ${A}_{12}^{ \acute{} }$ and ${A}_{21}^{ \acute{} }$, and the mass fractions of the two components, w_1_ and w_2_. The toxicological binary interaction coefficients, ${A}_{12}^{ \acute{} }$ and ${A}_{21}^{ \acute{} }$, are determined using the Mean Square Error method.

To apply the Van Laar-based model, the selection of the experimental point to calculate the fitting parameters (${A}_{12}^{ \acute{} }$ and ${A}_{21}^{ \acute{} }$) is a critical issue, and the accuracy of the estimation is expected to depend on the mass fractions of the compounds involved in the experiment. Therefore, a method has been developed to select these mass fractions using the CA model as an initial estimation of the fitting point. This method relies on the difference between the ideal prediction (Eq. 15) and the estimation with the CA model (Eq. 10), where the latter allows for the estimation of the mixture’s toxicity with only the knowledge of the individual toxicity of the compounds. The worst-case scenario occurs when the difference between the results of both models is the largest. In this case, the worst prediction is obtained, and it can be guaranteed that the estimation with the rest of the fitting points will be superior. Therefore, in order to maximize the absolute and relative differences between the CA model and the ideal predictions, denoted as $\triangle ={\left({{\rm{EC}}}_{50}\right)}_{{\rm{mix}},{\rm{CA}}}-{\left({{\rm{EC}}}_{50}\right)}_{{\rm{mix}}}^{* }$ and ${\triangle }_{r}=\frac{{\left({{\rm{EC}}}_{50}\right)}_{{\rm{mix}},{\rm{CA}}}-{\left({{\rm{EC}}}_{50}\right)}_{{\rm{mix}}}^{* }}{{\left({{\rm{EC}}}_{50}\right)}_{{\rm{mix}},{\rm{CA}}}}$, respectively, the derivatives of these differences with respect to the variable w_1_ have been analytically deduced, as shown in Eqs. 22 and 23.

22 $$\frac{{\rm{d}}\triangle }{{\rm{d}}{{\rm{w}}}_{1}}=\frac{{{({\rm{EC}}}_{50(2)}-{{\rm{EC}}}_{50(1)})}^{2}\left[{({\rm{EC}}}_{50(2)}-{{\rm{EC}}}_{50\left(1\right)}){{{\rm{w}}}_{1}}^{2}+2{{\rm{EC}}}_{50\left(1\right)}{{\rm{w}}}_{1}-{{\rm{EC}}}_{50\left(1\right)}\right]}{{\left[{({\rm{EC}}}_{50(2)}-{{\rm{EC}}}_{50\left(1\right)}){{\rm{w}}}_{1}+{{\rm{EC}}}_{50\left(1\right)}\right]}^{2}}$$

23 $$\frac{{\rm{d}}{\Delta }_{{\rm{r}}}}{{\rm{d}}{{\rm{w}}}_{1}}=-4{{\rm{w}}}_{1}+2+\frac{{{\rm{EC}}}_{50\left(1\right)}}{{{\rm{EC}}}_{50\left(2\right)}}\left(2{{\rm{w}}}_{1}-1\right)+\frac{{{\rm{EC}}}_{50\left(2\right)}}{{{\rm{EC}}}_{50\left(1\right)}}\left(2{{\rm{w}}}_{1}-1\right)$$

where w_1_ is the mass fraction of the least toxic compound, Δ corresponds to the absolute difference between the CA model and the ideal prediction,$\,{\Delta }_{r}$ gives the relative difference between the CA model and the ideal prediction, and EC_50(1)_ and EC_50(2)_ correspond to the individual EC_50_ of each compound.

To determine the value of w_1_ that maximizes the absolute and relative differences between the CA model and the ideal prediction, it is necessary to find the point where the respective derivative functions are zero. The expression for w_1_ that corresponds to the maximum absolute difference (${\left({{\rm{w}}}_{1,\max }\right)}_{\Delta }$) is given by Eq. 24, and the expression for w_1_ that corresponds to the maximum relative difference (${\left({{\rm{w}}}_{1,\max }\right)}_{{\Delta }_{r}}$) is given by Eq. 25.

24 $${\left({{\rm{w}}}_{1,\max }\right)}_{\Delta }=\pm \frac{\sqrt{{{\rm{EC}}}_{50\left(1\right)}{{\rm{EC}}}_{50\left(2\right)}}-{{\rm{EC}}}_{50\left(1\right)}}{{{\rm{EC}}}_{50(2)}-{{\rm{EC}}}_{50(1)}}$$

25 $${\left({{\rm{w}}}_{1,{\max}}\right)}_{{\Delta }_{r}}=\frac{1}{2}{\rm{if}}\left({{\rm{EC}}}_{50\left(1\right)}\cdot {\rm{EC}}_{50\left(2\right)}\right)\,\ne\, 0{\rm{;}}\left({{\rm{EC}}}_{50\left(1\right)}\,\ne\, {\rm{EC}}_{50\left(2\right)}\right)$$

## Results and discussion

### Experimental toxicity of EDA and ACM

The toxicity of the individual compounds (EDA and ACM) to *A. fischeri* was determined, before assessing their combined effects. The concentration-response curve of each chemical was fitted using the Weibull model. The achieved Weibull parameters, α and β, were obtained by minimizing the sum of square errors and are shown in Table S2.

**Fig. 1:**
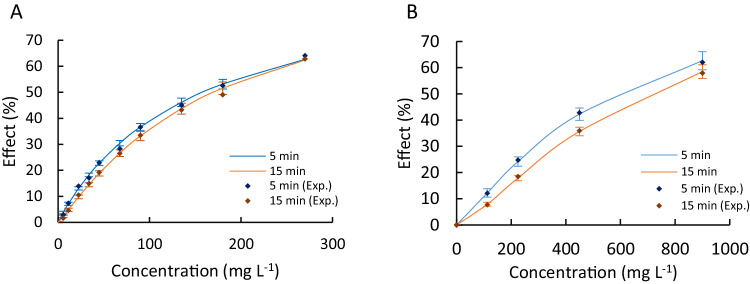
Concentration-response curves. **A** Edaravone. **B** Acetaminophen

In Fig. 1, the experimental data (dots) and the fitted concentration-response curves for EDA and ACM are presented. The EC_50_ values for EDA in the Microtox® acute toxicity test was determined to be 158 ± 10 mg/L and 170 ± 13 mg/L for exposure times of 5 min and 15 min, respectively, with a 95% confidence level. The EC_50_ values for ACM were 584 ± 55 mg/L and 697 ± 48 mg/L for exposure times of 5 min and 15 min, respectively, with a 95% confidence level. Additionally, a Student’s t-test was performed, revealing a statistically significant difference in the results between the 5-minute and 15-minute exposure times for both substances, with a confidence level of 95%.

As observed, the toxic effect of ACM and EDA on microorganisms decreases with increasing exposure time. In other words, a higher dose of a compound is required to produce the same effect at a longer exposure time. This phenomenon is more evident for ACM compared to EDA, potentially due to ACM’s greater susceptibility to degradation. Henschel et al. (1997) reported a 57% degradation, indicative of its near-readily biodegradability in an OECD 301 Ready Biodegradability Test. Moreover, Richardson and Bowron (1985) demonstrated Acetaminophen’s readiness for biodegradation following adaptation. Hence, the observed and statistically validated slightly difference in toxicity after 15 min may stem from biodegradation.

In accordance with previously published studies (Calleja et al. 1993; Henschel et al. 1997; Kim et al. 2007; Ortiz de García et al. 2014), the EC_50_ values of ACM obtained in our investigation are within the same order of magnitude. However, to the best of our knowledge, there are no previous ecotoxicological studies on EDA, making it impossible to make a similar comparison.

Additionally, based on the ecotoxicity values obtained, both EDA and ACM would fall under the “Non-toxic” category as per the Globally Harmonized System of Classification and Labeling of Chemicals (GHS). This means that neither of these compounds would pose a significant environmental threat if present individually.

### Experimental and estimated toxicity of binary mixtures of ACM and EDA under the CA and IA models

**Fig. 2:**
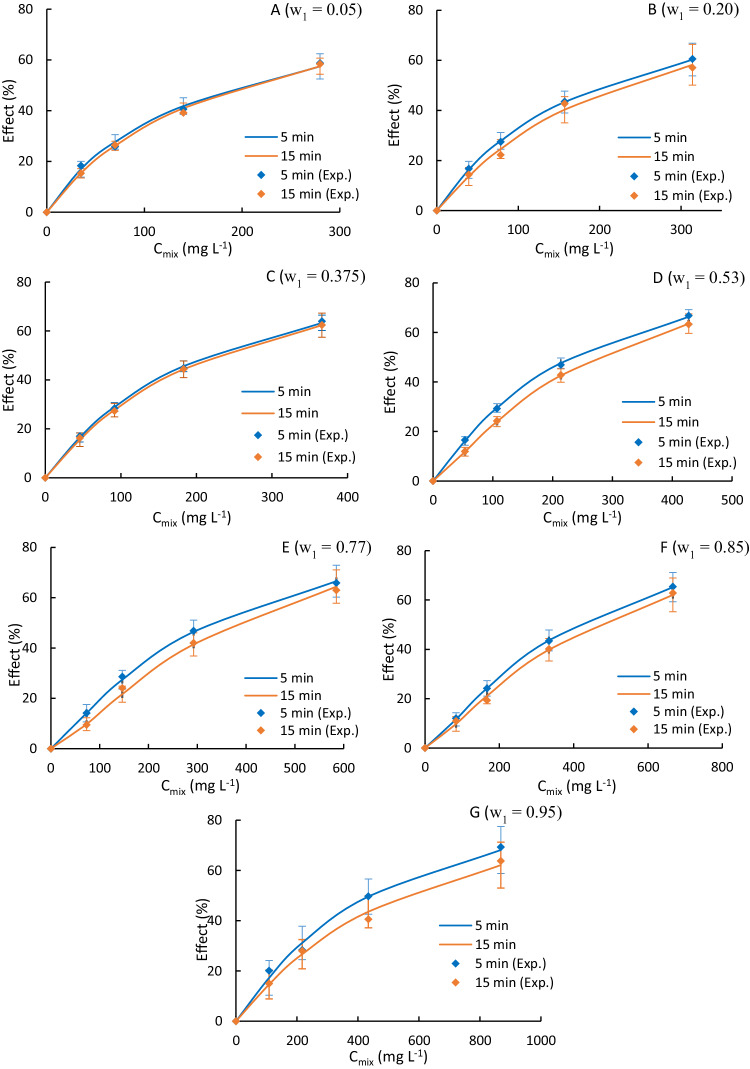
Microtox® Concentration-Response Curves of Binary Mixtures of Acetaminophen and Edaravone as a Function of Acetaminophen Mass Fraction (w_1_). Plots show the concentration-response curves of binary mixtures of Acetaminophen and Edaravone as a function of acetaminophen mass fraction (*w*_1_ = *w*_ACM_) for: **A** *w*_1_ = 0.05; **B** *w*_1_ = 0.20; **C** *w*_1_ = 0.375; **D** *w*_1_ = 0.53; **E** *w*_1_ = 0.77; **F** *w*_1_ = 0.85; **G** *w*_1_ = 0.95

Figure 2 shows the concentration-response curves obtained from the Microtox® bioluminescence assay for the aqueous solutions of binary mixtures with different mass fractions of ACM (w_1_) ranging from 0.05 to 0.95. This wide range of concentrations was chosen to validate the maximum difference method and to investigate the accuracy of the fitting point in the new Van Laar-based model that will be discussed later. Notably, the concentration-response curves obtained in this study will enable us to assess the combined effect of ACM and EDA on *A. fischeri*.

Experimental EC_50_ values for the binary mixtures of ACM and EDA were obtained from the concentration-response curves shown in Fig. 2, as was done previously for each compound individually. Table S3 summarizes the EC_50_ values obtained from the experimental data.

Regarding the toxicology models, the CA model allowed for an explicit solution to estimate the EC_50_ of the mixture using Eq. 10. However, for the IA model, an iterative process was necessary to complete the same estimation, as seen in Eq. 12. To apply the IA model, Concentration-Effect curves of each compound were fitted to the Weibull function (Eq. 13). The Weibull parameters, α and β, obtained through the minimization of the sum of square errors are presented in Table S2.

**Fig. 3:**
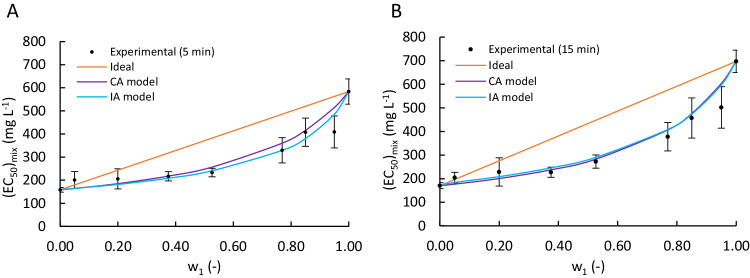
Experimental (dots) and estimated (lines) EC_50_ values of Acetaminophen-Edaravone binary mixtures as a function of Acetaminophen mass fraction (w_1_) and exposure time (**A**. 5 min, **B**. 15 min)

Figure 3 shows the toxicity estimations obtained using the CA and IA models. In comparison to the experimental results, both models demonstrated a strong predictive capability for this mixture of pharmaceuticals. This result contrasts with the findings of Trombini et al. (2016) for the copepod *Tisbe battagliai*, in which they observed the inability of the CA and IA models to accurately predict the toxicity of binary mixtures of pharmaceuticals (e.g., ACM, carbamazepine, diclofenac and ibuprofen).

Furthermore, a slight discrepancy can be observed between the IA and CA models for an exposure time of 5 min, with the IA model predicting greater toxicity than the CA model for w_1_ > 0.5. A similar discrepancy was reported by Villa et al. (2014) for Triclosan, methyl-triclosan and Triclocarban. However, in other studies such as Backhaus et al. (2000) (with mixtures of several PhACs, including antibiotics) or Faust et al. (2003) (using 16 different biocides), the opposite was observed. In the case of Backhaus et al. (2000) (which employed herbicides), it was suggested that the CA and IA models are equivalent under certain conditions: the Concentration-Effect curve of each compound must be described with the Weibull model, both curves must be parallel, and β must be approximately 2.3. It can be verified that these conditions are satisfied for ACM and EDA. Hence, both models may be equivalent, which explains the minimal difference in their toxicity estimation, especially for an exposure time of 15 min.

Additionally, it is observed that for solutions in which the mass fraction of ACM (w_1_) exceeds 0.375 and 0.2 (for exposure times of 5 and 15 min, respectively), the EC_50_ values are lower than those predicted by an additive model (i.e., CI < 1). This suggests that these binary mixtures of PhACs exhibit greater toxicity than expected from an additive model. In summary, these binary mixtures of PhACs induce synergistic effects. However, for a w_1_ lower than 0.375 (for exposure times of 5 or 15 min), the presence of ACM in the mixture reduces the toxic effect compared to an additive contribution (CI > 1), indicating an antagonistic effect (see Table S4). This finding is consistent with a previous study by Sung et al. (2014), which reported that a binary mixture of Acetaminophen-Ibuprofen at low ACM concentrations resulted in less toxicity than expected for a freshwater shrimp (*Neocaridina denticulata*).

Cedergreen et al. (2008) conducted a study on 98 different mixtures of pesticides and drugs using a variety of model organisms, including *A. fischeri*. Their results showed that synergistic deviations were rare, with only 6% of the mixtures showing an ecotoxicological synergy.

The study by Cedergreen et al. also assessed the accuracy of the CA and IA models in predicting ecotoxicological effects. One of the microorganisms used in the study was *A. fischeri*, for which the two models failed to describe around 60% of the tests, while approximately 15% of the tests could be described only by the IA model and 5% only by the CA model. Both models could describe the ecotoxicity of around 20% of the tests. Therefore, it is necessary to refine the estimations with other models that can better capture the potential complexity of the mechanisms of action.

At concentrations of ACM lower than 5% by mass, none of the models were able to predict the observed antagonistic effect, which highlights the models’ limitations in this range. However, both models provided good predictions of the experimental data, with a coefficient of determination greater than 0.92 in all cases (Table 1). Furthermore, the predictive power of both models was found to depend on the exposure time, with the CA model performing worse than the IA model for an exposure time of 5 min. Conversely, the CA model was more accurate than the IA model for an exposure time of 15 min. Backhaus et al.’s (2000) estimation of mixtures of several PhACs partially supports our results, as they showed that the IA model had greater predictive power than the CA model, which may be due to the different mechanisms of action of the compounds used.

**Table 1:** Performance evaluation of Concentration Addition (CA) and Independent Action (IA) models for Acetaminophen and Edaravone toxicity prediction in *Aliivibrio fischeri*: Error and R-squared values at 5- and 15-minutes exposure

	Error (%)		R^2^ (-)	
Model	5 min	15 min	5 min	15 min
CA	9.72	7.93	0.9679	0.9909
IA	8.91	9.19	0.9263	0.9718

### Bacterial toxicity of ACM and EDA binary mixtures estimated with a Van Laar-based model

**Fig. 4:**
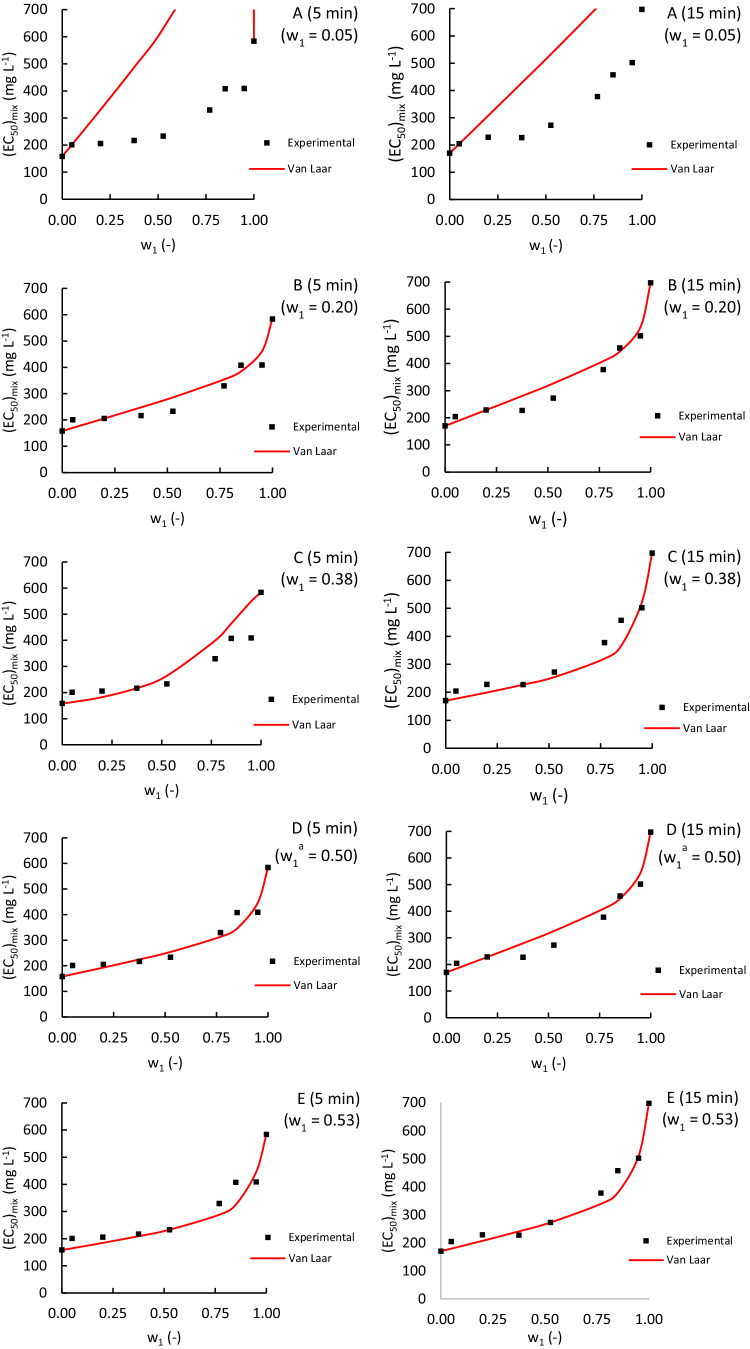

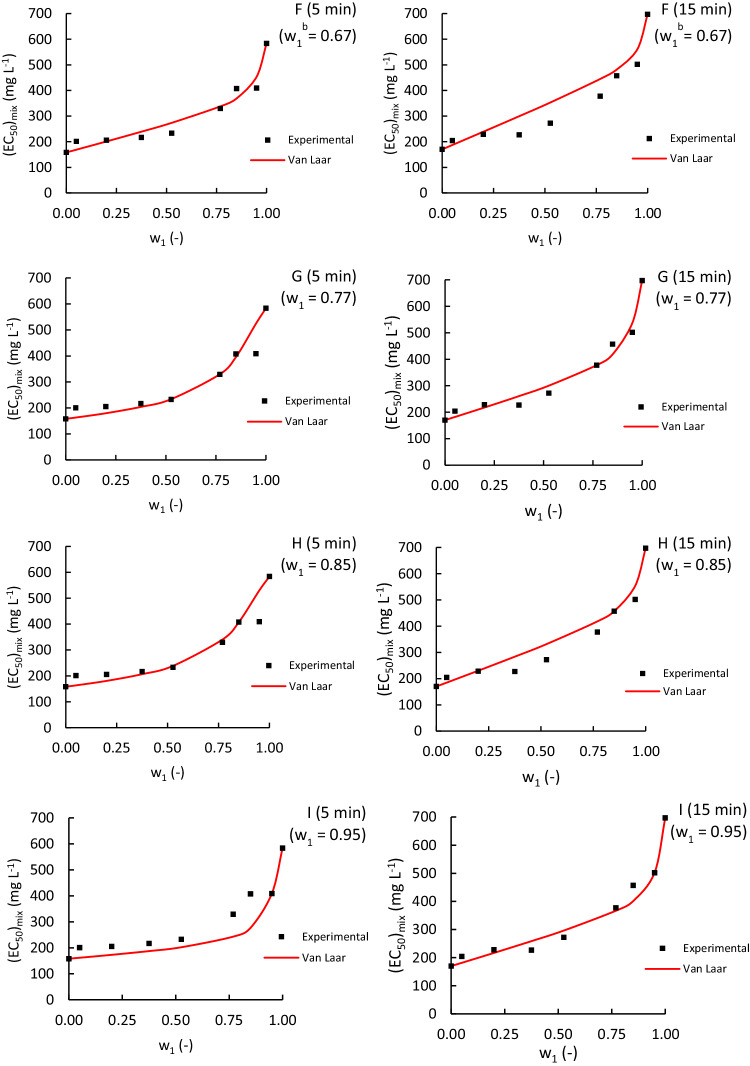
Comparison of experimental (dots) and Van Laar-based model estimated (red lines) (EC_50_)_mix_ values for binary mixtures of Acetaminophen and Edaravone. The Van Laar-based model lines were obtained using one experimental (EC_50_)_mix_ value for a mass fraction w_1_ of Acetaminophen (upper-right corner). ^a^Maximum relative difference between the CA model and the ideal prediction of ${\left({{\rm{EC}}}_{50}\right)}_{{\rm{mix}}}$. ^b^Maximum absolute difference between the CA model and the ideal prediction of ${\left({{\rm{EC}}}_{50}\right)}_{{\rm{mix}}}$ Plots show experimental data compared with predictions made using the Van Laar-based model line obtained using the experimental (EC_50_)_mix_ value of the binary mixture of Edaravone and Acetaminophen for the following Acetaminophen mass fraction (*w*_1_ = *w*_ACM_): **A** *w*_1_ = 0.05 for exposure times of 5 min (left plot) and 15 min (right plot); **B** *w*_1_ = 0.20 for exposure times of 5 min (left plot) and 15 min (right plot); **C** w_1_ = 0.38 for exposure times of 5 min (left plot) and 15 min (right plot); **D** *w*_1_ = 0.50 for exposure times of 5 min (left plot) and 15 min (right plot); **E** *w*_1_ = 0.53 for exposure times of 5 min (left plot) and 15 min (right plot); **F** *w*_1_ = 0.67 for exposure times of 5 min (left plot) and 15 min (right plot); **G** *w*_1_ = 0.77 for exposure times of 5 min (left plot) and 15 min (right plot); **H** *w*_1_ = 0.85 for exposure times of 5 min (left plot) and 15 min (right plot); **I** *w*_1_ = 0.95 for exposure times of 5 min (left plot) and 15 min (right plot)

Figure 4 shows that the Van Laar-based model provided reasonably good estimations for most cases of the ACM-EDA mixture, despite using fitting parameters determined with only one experimental data point. However, it is important to note that the model failed to detect the observed antagonistic effect at low ACM concentrations. Additionally, we could not compare our predictions with those of other researchers, as no previous studies have used a Van Laar-based model in ecotoxicology.

Table S5 presents Van Laar-based parameters and the coefficient of determination as a function of the selected fitting point. The model provides poor estimations for both low and high concentrations of ACM as the fitting point, while similar estimations are obtained for intermediate points regardless of the fitting point. The predictions obtained through the methods of maximum absolute and relative differences are similar to those of the intermediate points in the range. Therefore, the concentration chosen is irrelevant as long as it falls within the intermediate range.

**Fig. 5:**
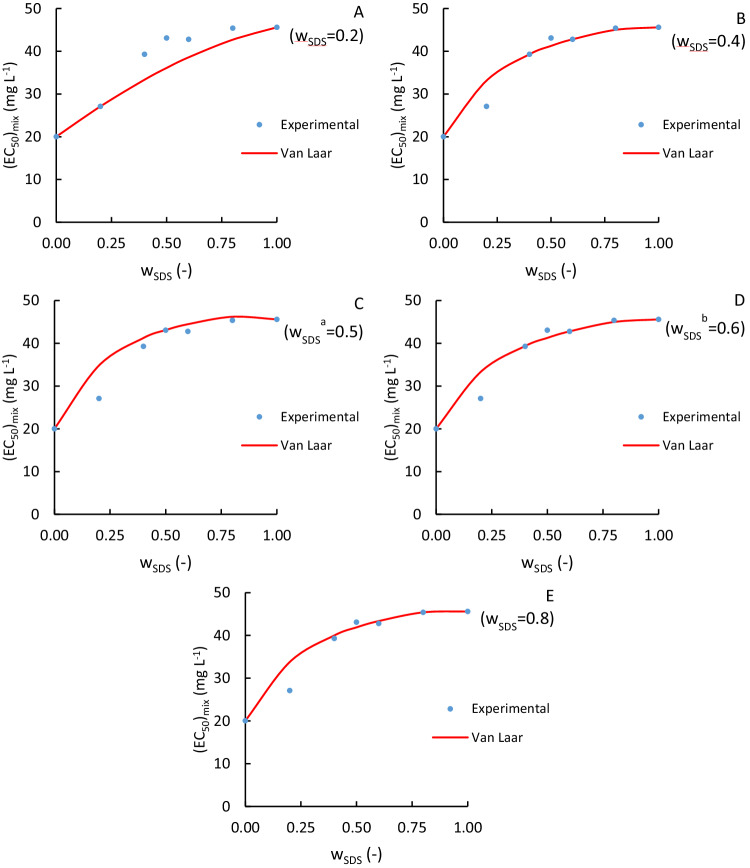
Comparison of experimental (dots) and Van Laar-based model estimated (red lines) ${\left({{\rm{EC}}}_{50}\right)}_{{\rm{mix}}}$ values for mixtures of SDS and GA (Boillot and Perrodin 2008). The Van Laar-based model lines were obtained using one experimental ${\left({{\rm{EC}}}_{50}\right)}_{{\rm{mix}}}$ value for a mass fraction w_SDS_ (upper-right corner). ^a^Maximum relative difference between the CA model and the ideal prediction of ${\left({{\rm{EC}}}_{50}\right)}_{{\rm{mix}}}$. ^b^Maximum absolute difference between the CA model and the ideal prediction of ${\left({{\rm{EC}}}_{50}\right)}_{{\rm{mix}}}$. The plots show, similar to Fig. 4, experimental data compared with predictions made using the Van Laar-based model line obtained using the experimental (EC50)mix value for: **A** wSDS = 0.2; **B** wSDS = 0.4; **C** wSDS = 0.6; **D** wSDS = 0.8; **E** wSDS = 1.0.

To validate the developed model, experimental data of another binary mixture was extracted from the literature. Boillot and Perrodin (2008) conducted acute toxicity (EC_50_-24 h) bioassays on *Daphnia magna*, a planktonic crustacean, for binary mixtures of glutaraldehyde (GA) and different anionic surfactants, such as sodium dodecyl sulfate (SDS). The binary mixtures of GA and SDS were used to validate the new Van Laar-based model proposed in this study. Figure 5 shows the experimental data from Boillot and Perrodin (2008) and the estimations performed with the Van Laar-based model proposed in this study.

Table 2 shows the achieved Van Laar parameters (${A}_{12}^{ \acute{} }$ and ${A}_{21}^{ \acute{} }$) and the coefficient of determination as a function of the mass fraction of SDS to evaluate the accuracy obtained. Overall, a satisfactory coefficient of determination is obtained. However, a comparison between the Van Laar-based model estimation and the one obtained by Boillot and Perrodin (2008) with the Toxicity Index model, which is another commonly used model for predicting mixture toxicity, cannot be made as the latter publication does not provide numerical data on the achieved accuracy.

**Table 2:** Van Laar-based model parameters and coefficient of determination for fitting the SDS-GA mixture at various SDS mass fractions

w_SDS_ (-)	A'_12_ (-)	A'_21_ (-)	Error (%)	R^2^ (-)
0.20	0.2264	0.5697	9.44	0.9170
0.40	0.9360	0.9054	5.47	0.9426
0.50^a^	1.0491	1.1090	7.82	0.9255
0.60^b^	0.9677	0.8968	5.64	0.9393
0.80	0.9930	0.9687	6.12	0.9363

^a^Maximum relative difference between the CA model and the ideal prediction of ${\left({{\rm{EC}}}_{50}\right)}_{{\rm{mix}}}$

^b^Maximum absolute difference between the CA model and the ideal prediction of ${\left({{\rm{EC}}}_{50}\right)}_{{\rm{mix}}}$

## Conclusions

PhACs and their metabolites are acknowledged as emerging pollutants in ecosystems. Literature has demonstrated interactions between PhACs. Hence, it is important to determine their combined effect to avoid underestimating their toxicity. This study initially revealed that ACM and EDA, two PhACs, are classified as “Non-toxic” according to the GHS classification system. Additionally, the obtained results for ACM are consistent with literature data, which report EC_50_ > 100 mg L^−1^. However, no well documented ecotoxicological studies have been associated with EDA to date, to the best of our knowledge.

Regarding the study of ACM/EDA binary mixtures, it has been determined that there is a synergetic effect at ACM mass concentrations higher than 37.5 and 20% in the mixture, for exposure times of 5 and 15 min, respectively. However, at lower ACM mass concentrations, an antagonistic action is observed. In terms of the prediction accuracy of the CA and IA models, they were found to provide satisfactory estimation power.

On the other hand, a semi-empirical Van Laar-based model was developed to estimate the bacterial toxicity of the ACM/EDA mixture. The model provided good predictive results for the experimental data and was validated with literature experiments, showing acceptable bacterial toxicity estimation. The estimation accuracy of the model was found to depend on the selected testing point for the fitting. Poor estimations were obtained when low and high concentrations of ACM or EDA were used, whereas the predictive power improved when intermediate points were chosen.

### Supplementary information

Supplementary Information
