# Supplementary material for: Bacterial toxicity of Acetaminophen and Edaravone, and their binary mixtures: experimental and predicted values using traditional and novel Van Laar-based models
Source: Ecotoxicology. 2024 Jun 29;33(7):722–36. doi: 10.1007/s10646-024-02772-w (PMC11358354; doi:10.1007/s10646-024-02772-w)
Supplement: Supplementary file 1 — Supplementary Information [file 10646_2024_2772_MOESM1_ESM.docx]

**Supplementary material**

**Table S1**. Chemical structure and characteristics of Edaravone and Acetaminophen (NCBI, 2021a; NCBI, 2021b)

| Compounds | CAS number | Molecular formula | Molecular weight (g/mol) | Water solubility (mg/L) | log K_ow_ | pK_a_ | Chemical Structure |
| --- | --- | --- | --- | --- | --- | --- | --- |
| 3-Methyl-1-phenyl-2-pyrazolin-5-one  (Edaravone) | 89-25-8 | C_10_H_10_N_2_O | 174.20 | <1000 | 2.56 | 7.0 | 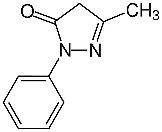 |
| N- (4-Hydroxyphenyl) acetamide  (Acetaminophen) | 103-90-2 | [C_8_H_9_NO_2_](https://pubchem.ncbi.nlm.nih.gov/search/#collection=compounds&query_type=mf&query=C8H9NO2&sort=mw&sort_dir=asc) | 151.16 | 14 000 | 0.46 | 9.38 | 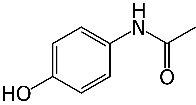 |

**Table S2**. Weibull fitting parameters for the IA model

| **PhAC** | **Weibull parameters** | **Value** | | **R^2^** |
| --- | --- | --- | --- | --- |
|  |  | **5 min** | **15 min** | **(-)** |
| Acetaminophen | α | -6.23 | -7.34 | 0.9865 |
|  | β | 2.11 | 2.44 |  |
| Edaravone | α | -4.43 | -4.81 | 0.9820 |
|  | β | 1.84 | 1.98 |  |

**Table S3**. Experimental and ideal EC_50_ values of Acetaminophen-Edaravone binary mixtures

|  |  |  | | **Experimental** | | | |  | |  | | | **Ideal** | | | |  |
| --- | --- | --- | --- | --- | --- | --- | --- | --- | --- | --- | --- | --- | --- | --- | --- | --- | --- |
| **Mass fraction** | | **5 min** | | | | **15 min** | | | | | **5 min** | | | | **15 min** | | |
| **w_1_**  **(-)** | **w_2_**  **(-)** | | **EC_50_**  **(mg L^-1^)** | |  | | **EC_50_**  **(mg L^-1^)** | |  | | | **EC_50_**  **(mg L^-1^)** | |  | | **EC_50_**  **(mg L^-1^)** |  |
| 0.00 | 1.00 | 158 ± 10 | | |  | | 170 ± 13 | |  | | | 158.17 | |  | | 170.10 |  |
| 0.05 | 0.95 | 201 ± 37 | | |  | | 204 ± 23 | |  | | | 179.45 | |  | | 196.47 |  |
| 0.20 | 0.80 | 206 ± 44 | | |  | | 229 ± 60 | |  | | | 243.30 | |  | | 275.55 |  |
| 0.375 | 0.625 | 217 ± 21 | | |  | | 227 ± 22 | |  | | | 317.78 | |  | | 367.82 |  |
| 0.53 | 0.47 | 233 ± 19 | | |  | | 272 ± 29 | |  | | | 382.18 | |  | | 447.60 |  |
| 0.77 | 0.23 | 329 ± 55 | | |  | | 378 ± 60 | |  | | | 485.57 | |  | | 575.67 |  |
| 0.85 | 0.15 | 408 ± 61 | | |  | | 457 ± 85 | |  | | | 519.94 | |  | | 618.26 |  |
| 0.95 | 0.05 | 409 ± 69 | | |  | | 502 ± 88 | |  | | | 562.50 | |  | | 670.98 |  |
| 1.00 | 0.00 | 584 ± 55 | | |  | | 697 ± 48 | |  | | | 583.78 | |  | | 697.34 |  |

**Table S4**. Combination Indexes (CI) of Acetaminophen-Edaravone binary mixtures for exposure times of 5 and 15 minutes

| **Mass fraction** | | **CI** | |
| --- | --- | --- | --- |
| **w_1_**  **(-)** | **w_2_**  **(-)** | **5 min**  **(-)** | **15 min**  **(-)** |
| 0.00 | 1.00 | 1.00 | 1.00 |
| 0.05 | 0.95 | 1.22 | 1.16 |
| 0.20 | 0.80 | 1.11 | 1.14 |
| 0.38 | 0.63 | 1.00 | 0.96 |
| 0.53 | 0.47 | 0.91 | 0.96 |
| 0.77 | 0.23 | 0.91 | 0.93 |
| 0.85 | 0.15 | 0.98 | 0.96 |
| 0.95 | 0.05 | 0.80 | 0.83 |
| 1.00 | 0.00 | 1.00 | 1.00 |

**Table S5**. Van Laar-based model parameters and coefficient of determination for Acetaminophen-Edaravone binary mixtures as a function of selected fitting point

|  | **5 minutes** | | | | **15 minutes** | | | |
| --- | --- | --- | --- | --- | --- | --- | --- | --- |
| **w_1_**  **(-)** | **A’_12_**  **(-)** | **A’_21_**  **(-)** | **Error (%)** | **R^2^**  **(-)** | **A’_12_**  **(-)** | **A’_21_**  **(-)** | **Error**  **(%)** | **R^2^**  **(-)** |
| 0.05 | 0.5518 | -16.6575 | 1163.9 | 0.3587 | 0.2052 | -21.1090 | 85.9 | 0.9206 |
| 0.20 | -0.3923 | -16.8112 | 11.2 | 0.8938 | -0.4145 | -21.2168 | 21.7 | 0.9399 |
| 0.38 | -0.8411 | -2.2202 | 16.1 | 0.9630 | -0.8029 | -18.3782 | 9.9 | 0.9106 |
| 0.50ª | -0.5685 | -17.7788 | 9.3 | 0.8816 | -0.4221 | -17.5984 | 22.1 | 0.9429 |
| 0.53 | -0.7113 | -16.8790 | 10.6 | 0.8552 | -0.6849 | -21.3292 | 10.6 | 0.9307 |
| 0.67^b^ | -0.4558 | -17.5520 | 9.9 | 0.8924 | -0.3081 | -17.1587 | 29.0 | 0.9315 |
| 0.77 | -0.8291 | -5.5990 | 9.8 | 0.9167 | -0.5431 | -17.1320 | 9.9 | 0.9444 |
| 0.85 | -0.8155 | -4.8791 | 10.1 | 0.9281 | -0.3973 | -15.7649 | 23.9 | 0.9426 |
| 0.95 | -0.9345 | -23.6193 | 17.2 | 0.7830 | -0.5510 | -25.9081 | 13.9 | 0.9424 |

^a^Maximum relative difference between the CA model and the ideal prediction of $\left( \mathrm{EC}_{50} \right)_{\mathrm{mix}}$.

^b^Maximum absolute difference between the CA model and the ideal prediction of $\left( \mathrm{EC}_{50} \right)_{\mathrm{mix}}$.
